# Face Attribute Estimation Using Multi-Task Convolutional Neural Network

**DOI:** 10.3390/jimaging8040105

**Published:** 2022-04-10

**Authors:** Hiroya Kawai, Koichi Ito, Takafumi Aoki

**Affiliations:** Graduate School of Information Sciences, Tohoku University, 6-6-05, Aramaki Aza Aoba, Sendai 9808579, Japan; aoki@ecei.tohoku.ac.jp

**Keywords:** face attribute estimation, CNN, multi-task learning, deep learning, biometrics

## Abstract

Face attribute estimation can be used for improving the accuracy of face recognition, customer analysis in marketing, image retrieval, video surveillance, and criminal investigation. The major methods for face attribute estimation are based on Convolutional Neural Networks (CNNs) that solve face attribute estimation as a multiple two-class classification problem. Although one feature extractor should be used for each attribute to explore the accuracy of attribute estimation, in most cases, one feature extractor is shared to estimate all face attributes for the parameter efficiency. This paper proposes a face attribute estimation method using Merged Multi-CNN (MM-CNN) to automatically optimize CNN structures for solving multiple binary classification problems to improve parameter efficiency and accuracy in face attribute estimation. We also propose a parameter reduction method called Convolutionalization for Parameter Reduction (CPR), which removes all fully connected layers from MM-CNNs. Through a set of experiments using the CelebA and LFW-a datasets, we demonstrate that MM-CNN with CPR exhibits higher efficiency of face attribute estimation in terms of estimation accuracy and the number of weight parameters than conventional methods.

## 1. Introduction

Face recognition is one of the most attractive topics in biometrics and computer vision because of its convenience, hygiene, and low cost, since face images can be acquired in a contactless manner without requiring any special equipment [1]. Face recognition is in great demand as personal authentication for smartphones, security gates, payment services, communication robots, etc. due to its advantages. Although the explosive development of Convolutional Neural Networks (CNNs) has dramatically improved the accuracy of face recognition, face recognition still faces the problem that its accuracy is significantly degraded by changes in pose, facial expression, motion, illumination, and resolution. To address the great demand for face recognition, further improvements in its performance have been investigated. There are two approaches to improve the performance of face recognition: a direct approach to improve the face recognition method and an indirect approach to improve the performance by adding other factors to the face recognition method. In this paper, we focus on face attribute estimation, which is an indirect approach, in the sense that it can be used not only for improving the accuracy of face recognition but also for customer analysis in marketing, image retrieval, video surveillance, and criminal investigation [2,3].

A face has a wide variety of biological features, including age, gender, hair color, hairstyle, mouth size, nose height, etc. These facial features, called face attributes, cannot be used for personal identification on their own; however, they can be used together for rough personal identification. This use of biometric traits is known as soft biometrics, in contrast to hard biometrics, where a single biometric trait such as fingerprint, iris, or face can be used for personal identification. For example, the recognition accuracy of face recognition methods can be improved by combining general face features with face attributes [4,5]. The processing time of face recognition can be reduced by prescreening using face attributes.

**Figure 1:**
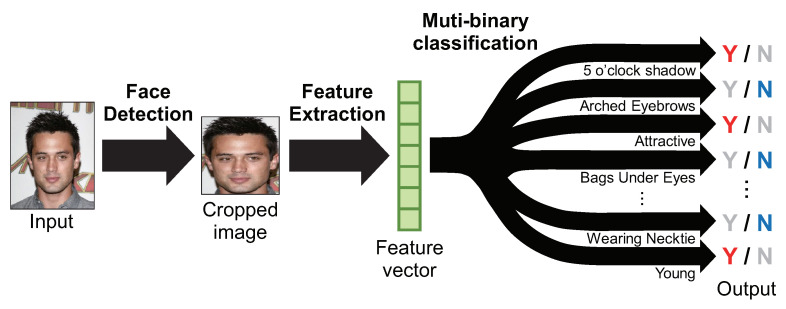
A typical processing flow of face attribute estimation. Face attribute estimation consists of multiple two-class classification problems. First, a face region is detected from a face image, and features are extracted. Then, the features are input to a discriminator for each attribute, and the presence or absence of the attribute is estimated.

Face attribute estimation can be regarded as a multiple binary classification problem, as shown in Figure 1; that is, it is a problem of estimating whether a face has or does not have the attribute. Face attributes have multiple names depending on their color and shape, such as hair, or are expressed numerically, such as age. To deal with face attribute estimation as a binary classification problem, for example, hair can be decomposed into several classes such as black hair, blond hair, brown hair, and gray hair, and age can be simplified to young. Face attribute estimation consists of three processes: face detection, feature extraction, and classification [3,6]. Among these processes, feature extraction is the most important process, since it has the greatest impact on the estimation accuracy.

Traditional methods utilize hand-crafted features such as Local Binary Patterns (LBP) [7] in feature extraction. The LBP-based methods can estimate attributes from only one face image, since they do not require any training process; however, their estimation accuracy is quite low, since LBP cannot handle a wide variety of face attributes. CNN-based approaches have recently become the most popular approach for face attribute estimation, since CNN has made a significant impact on image recognition. Although one feature extractor should be used for each attribute to explore the accuracy of attribute estimation, in most cases, one feature extractor is shared to estimate all face attributes for the parameter efficiency [2,8,9,10,11,12,13,14]. To achieve both high parameter efficiency and high estimation accuracy, it is necessary to design CNN consisting of multiple layers such as convolution and pooling layers to extract the optimal features for each attribute. Several methods have been proposed to improve the accuracy of face attribute estimation by appropriately sharing the layers of CNNs [2,13,14,15]. In those methods, the manual grouping and clustering of face attributes were used to share layers of CNNs based on grouping. Manual grouping is not only time consuming but also arbitrary, and simple attribute clustering is not always effective for attribute estimation.

In this paper, we propose a method to automatically optimize CNN structures for solving multiple binary classification problems in order to improve the processing efficiency and accuracy in face attribute estimation. The basic structure of CNN used in the proposed method, which is called Merged Multi-CNN (MM-CNN), consists of a large number of convolution blocks regularly located in the depth and width directions, which are connected to each other at each depth by merging layers. MM-CNN is automatically optimized for face attribute estimation by introducing trainable weight parameters for each merging layer between blocks. We also propose a parameter reduction method called Convolutionalization for Parameter Reduction (CPR), which removes all fully connected layers from MM-CNN. Through a set of experiments to evaluate the performance on two public datasets, Large-scale CelebFaces Attributes dataset (CelebA) [9] and Labeled Faces in the Wild-a dataset (LFW-a) [16], we demonstrate that MM-CNN can estimate face attributes with high accuracy using CNN with fewer weight parameters than conventional methods. This paper is a full version of our initial study [17] with a detailed description of the proposed method, a survey of recent works, and performance comparison. The contributions of this paper can be summarized as follows:

- We propose a novel CNN architecture, MM-CNN, specifically designed for multi-task processing; and
- We also propose CPR, which significantly reduces the parameters of CNN by removing fully connected layers.

## 2. Related Work

The conventional methods for face attribute estimation are summarized in Table 1. These methods can be categorized as Support Vector Machine (SVM), CNN, and others depending on the type of classifier. In the following, we give an overview of the conventional methods for each type of classifier.

The first type of methods employs SVM as classifiers, which are the earliest methods for face attribute estimation [6,8,9,10]. SVM is a machine learning method to determine the decision boundaries for separating classes in feature space. Kumar et al. [6] proposed one of the famous face attribute estimation methods using handcrafted local features. This method extracts pixel values from grayscale, RGB, and HSV color spaces, edge magnitude, and orientation as features and classifies them into each face attribute using SVM. After this work, most of the methods have employed CNN-based feature extractors due to its excellent performance on image recognition. Zhang et al. [8] proposed Pose Aligned Networks for Deep Attribute modeling (PANDA), which consists of feature extraction by CNNs with poselet detection and attribute prediction by a linear SVM for each attribute. Liu et al. [9] proposed two CNN architectures: LNet for face localization and ANet for face attribute prediction with a linear SVM for each attribute. Zhong et al. [10] extracted features using FaceNet [18] or VGG-16 [19] and predicted attributes using a linear SVM.

The second type of methods employs neural networks as classifier [2,12,13,14,15,21,23,25], where most methods employ a single CNN to complete feature extraction and classification as a multi-task CNN. Wang et al. [12] proposed a GoogLeNet-like network architecture consisting of three CNNs for face recognition, weather prediction, and location estimation. Face attributes are estimated from concatenated features in the fully connected layers. Hand et al. [2] proposed Multi-task deep Convolutional Neural Network (MCNN) with an AUXiliary network (MCNN-AUX). They separate the 40 face attributes into six or nine groups based on facial parts, and they extract features for each attribute group. Auxiliary network, which finally estimates face attributes based on the estimation results of the multi-task CNN, is added. Cao et al. [15] proposed Partially Shared Multi-task CNN (PS-MCNN). They separate the 40 face attributes into four groups: upper, middle, lower, and whole images, based on the position of each attribute in the face. The PS-MCNN aggregates the features extracted by the network for each group and estimates their attributes using a classifier consisting of fully connected layers. Gao et al. [13] proposed three small multi-task CNNs: ATNet, ATNet_G, and ATNet_GT. Although these approaches are similar to MCNN, CNNs are desinged according to multiple clusters obtained by classifying face attributes using the k-means algorithm. Han et al. [14] proposed a multi-label classification method using original labels determined by their own rule in light of correlation among face attributes. They separate the attributes into eight groups—one group related to the whole face and seven groups related to each facial parts—and design a special classifier architecture with multiple one output for each group. Fukui et al. [21] proposed Attention Branch Networks (ABN), which is a sort of general-purpose CNN with attention to features. ABN consists of two branches: an attention branch for generating a visualization map and a perception branch for classification. They demonstrated that the attention mechanism with a visualization map is effective for estimating face attributes. Bhattarai et al. [23] proposed a new loss function based on a continuous label, which is generated by word2vec [24] based on 40 face attributes labels written in text. Chen et al. [25] proposed a Hard Parameter Sharing-Channel Split network (HPS-CS) consisting of normal and group convolution layers.

The third type of methods employs other classifiers [11,27,28]. Huang et al. proposed Large Margin Local Embedding (LMLE)-kNN [11] and Cluster-based LMLE (CLMLE) [27]. They focused on the class imbalance of face attribute labels and proposed a learning method that takes into account the distance between small clusters generated for each class. In LMLE-kNN and CLMLE, DeepID2 [26] and ResNet-like CNN [29] are used for feature extraction, respectively. Ehrlich et al. [28] proposed Multi-Task Restricted Boltzmann Machines (MT-RBMs) with Principal Component Analysis (PCA).

Our approach is similar to MCNN [2], PS-MCNN [15], and ATNet [13]. Although the relationships among facial attributes are hierarchical and complex, these methods use manual or non-hierarchical clustering to make a preliminary set of groups of facial attributes. On the other hand, our approach automatically optimizes the network parameters by recognizing the relationships among face attributes during the training of CNN.

## 3. Fundamentals of Face Attributes

**Figure 2:**
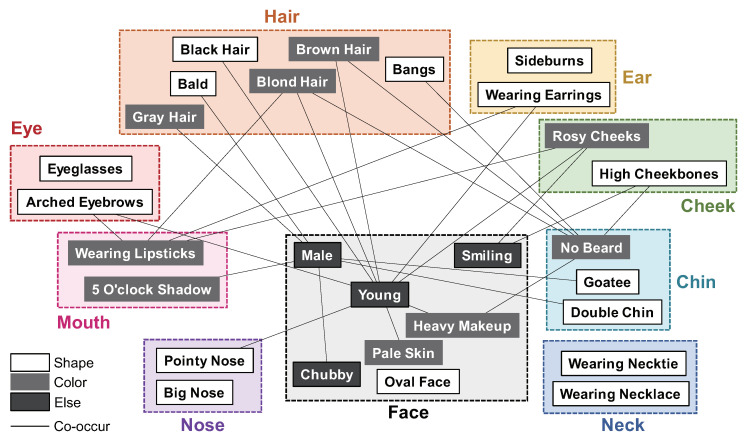
Example of illustrating the relationship among face attributes based on (i) commonality of facial parts, (ii) co-occurrence, and (iii) color, shape, and texture.

In this section, we give fundamental observations about the face attributes that we focus on in this paper. We use the 40 face attributes defined in CelebA [9], as shown in Table 2. CelebA is a large-scale dataset of face attributes that has been used for the training and performance evaluation of major face attribute estimation methods. In this paper, for convenience, each attribute is assigned an index number from 1 to 40, as shown in Table 2. Most of the attributes in CelebA are defined on the biological characteristics, while some are defined by whether the person wears ornaments such as glasses and earrings. These face attributes can be classified into groups based on the following relations: (i) commonality of facial parts, (ii) co-occurrence, and (iii) color, shape, and texture. Figure 2 shows an example of illustrating the relationship among face attributes based on relations (i)–(iii). In the following, we discuss the details of each relation.

**(i) Commonality of facial parts**—For face attribute labels, the most obvious relationship is based on the organs, that is, the facial parts included in the face. For example, Black Hair (9) and Wavy Hair (34) are attributes related to “hair,” Arched Eyebrows (2) and Narrow Eyes (24) are attributes related to “eyes,” and Big Nose (8) and Pointy Nose (28) are attributes related to “nose.” Note that the attribute labels such as Male (21), Attractive (3), and Young (40) are assigned to “face” in Figure 2, since they are based on the features of the entire face.

**Figure 3:**
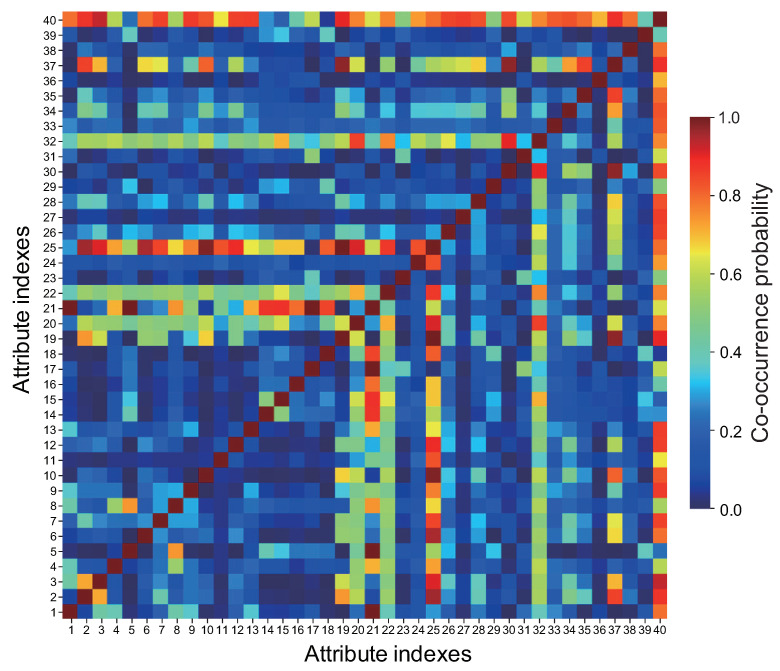
Color map visualizing the co-occurrence probabilities of 40 face attributes in CelebA.

**(ii) Co-occurrence**—Some attributes have co-occurrence, since they can appear simultaneously. Figure 3 shows a color map visualizing the co-occurrence probabilities of 40 face attributes in CelebA. The co-occurrence probability of two face attributes indicates the ratio of face images assigned with those two attributes. The face attributes with the highest co-occurrence probability are related to gender. Male (21) has a high probability of attributes such as 5 O’Clock Shadow (1), Bald (5), and Goatee (17), while female has a high probability of attributes such as Arched Eyebrows (2) and Heavy Makeup (19), where female means the face image without the Male (21) assignment. Exceptions are the co-occurrence of Smiling (32) with High Cheekbones (20) and Rosy Cheeks (30) for facial expressions, and Young (40) with Rosy Cheeks (30) for age. The co-occurrence of face attributes has a positive correlation in most cases, while there are some cases that have a negative correlation. For example, Gray Hair (18) symbolizing “aging” shows a high negative correlation with Young (40) and 5 O’Clock Shadow (1). No Beard (25) and Sideburns (31) also show a high negative correlation. We guess that Sideburns (31) is assigned a label as part of the beard in CelebA. However, note that such correlations between face attributes depend on the dataset. In Figure 3, Blond Hair (10) and No Beard (25) have high co-occurrence probability, while Black Hair (9) and No Beard (25) have low co-occurrence probability. This fact indicates that most of the females in CelebA have blond hair rather than black hair. CelebA consists mainly of Western celebrities and a very small number of Asian celebrities. Thus, the correlation of facial attributes strongly depends on ethnicity and gender.

**(iii) Color or shape or texture**—Most face attributes are related to either color, shape, or texture, except for abstract attributes such as age and gender. Color-related attributes include Black Hair (9), Blond Hair (10), Brown Hair (12), Gray Hair (18), Bags Under Eyes (4), Pale Skin (27), and Rosy Cheeks (30), shape-related attributes include Straight Hair (33) and Wavy Hair (34), Chubby (14), and Oval Face (26), and texture-related attributes include Blurry (11), Eyeglasses (16), and Heavy Makeup (19). The 5 O’Clock Shadow (1) and No Beard (25) attributes are related to both color and shape.

It is important to consider the above relationships among face attributes for estimating face attributes using multi-task CNN. In multi-task CNN, sharing feature extractors for face attributes with strong relationships can improve the estimation accuracy and reduce computational cost and memory consumption. There are complex relationships among face attributes, and it is difficult to manually design the optimal network architectures that takes them into account. To address this problem, in this paper, we propose a method to automatically optimize multi-task CNN for face attribute estimation.

## 4. Merged Multi-Convolutional Neural Network for Face Attribute Estimation

In this section, we describe the details of the Merged Multi-Convolutional Neural Network (MM-CNN) for face attribute estimation proposed in this paper.

### 4.1. Network Architecture of MM-CNN

**Figure 4:**
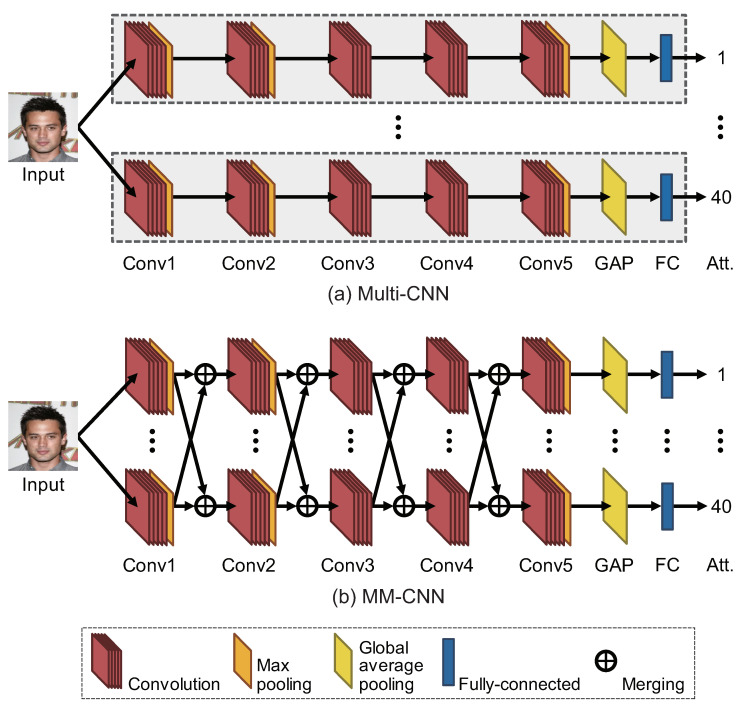
Overview of network architectures for (**a**) Multi-CNN and (**b**) MM-CNN.

We describe the network architecture of MM-CNN. First, we consider Multi-CNN that estimates attributes by inputting a face image to a small CNN for each attribute, as shown in Figure 4a. One small CNN is designed based on AlexNet [20], which consists of five convolution blocks and one fully connected layer. Note that the following points are different from the original AlexNet. In Conv1, the kernel size of convolution is changed to 7 × 7 from 11 × 11. In Conv2, the stride of convolution is changed to 1 from 2. All the normalization layers are replaced by the batch normalization layer [30]. The number of output channels in Conv5 is set to 1000, and the output of Conv5 is input to the Global Average Pooling (GAP) [31] layer. In the case of estimating 40 attributes, 40 single CNNs are set up in parallel, as shown in Figure 4a, with each CNN estimating one attribute. In this paper, the number of CNNs set in parallel is called “parallels”. Then, we design MM-CNN based on Multi-CNN as shown in Figure 4b. In MM-CNN, a unique layer called the merging layer is inserted after every convolutional block except Conv5. All the convolution blocks are connected to the merging layer for each stage, and their outputs are merged individually. The details of the merging layer are described below.

### 4.2. Merging Layer in MM-CNN

**Figure 5:**
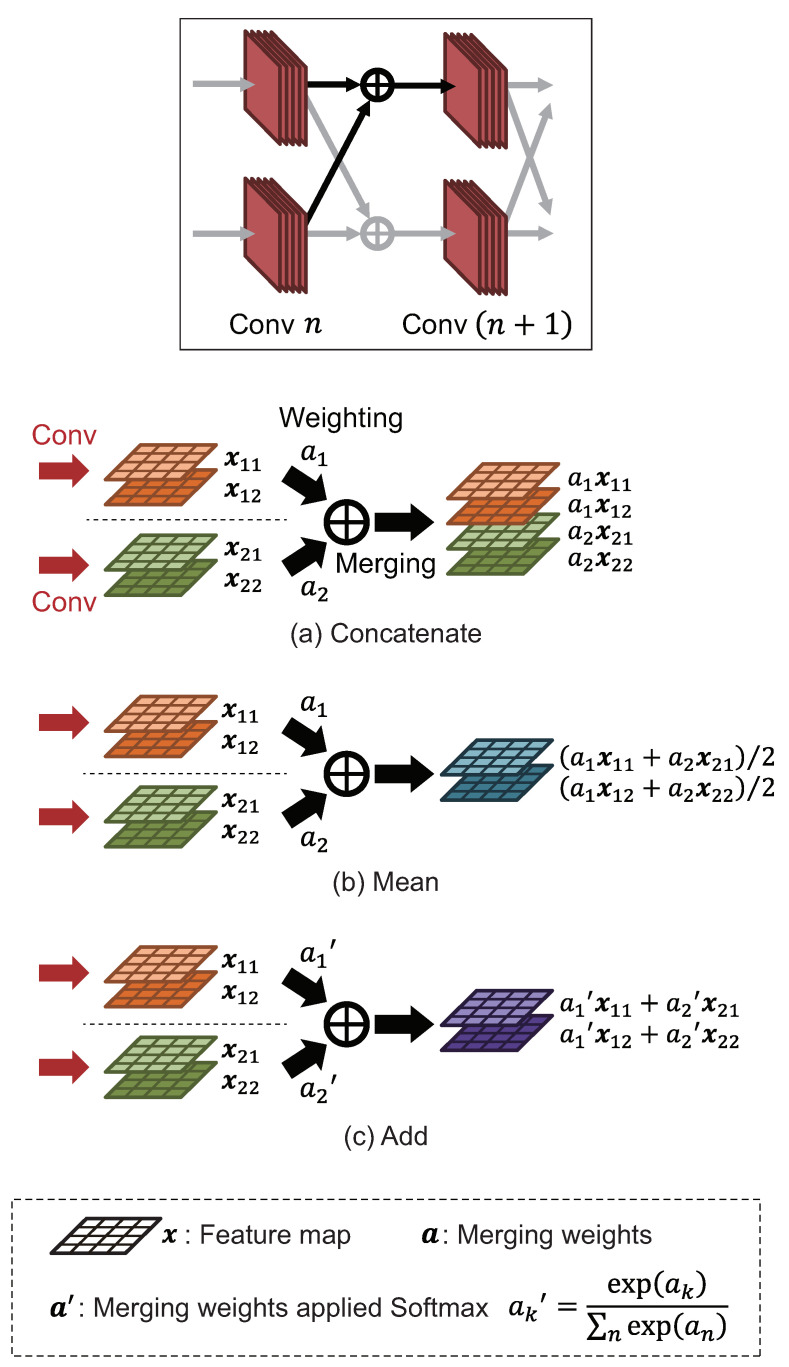
Overview of 3 types of merging function used in MM-CNN. For simplification, both the number of parallels and output channels of convolution layers are set to 2 in this figure.

The role of the merging layer is to merge multiple inputs into one, and a trainable weight parameter for merging is assigned to each input. The initial values of all the weight parameters are set to 1.0 unless otherwise specified. In the merging layer, the inputs are merged after weighting similarly to the fully connected layer. We consider three types of merging weighted inputs in the merging layer: Concat, Add, and Mean. In the following, we refer to these three types of merging as merging functions. An overview of each merging function is shown in Figure 5. In Concat, the weighted inputs are concatenated in the channel direction. In Mean, the weighted inputs are averaged for each channel. In Add, the weighted inputs are added for each channel. Since the value of the output feature map becomes extremely large if the weighted inputs are simply added, the weight $a^{\prime }$ is used by applying a softmax function to the weights a before weighting. Which merging function to use needs to be decided before training MM-CNN.

### 4.3. Convolutionalization for Parameter Reduction (CPR)

MM-CNN consists of the same number of CNNs as attributes; thus, it has a huge number of weight parameters. The larger the size of CNN, the higher its performance may be; however, the higher its computational cost and memory consumption. It is not practical to use such large CNNs due to the limited computational resources available on the device such as cell phones and PCs. Therefore, we introduce two approaches to reduce the number of weight parameters to be trained in MM-CNN.

The first approach is to control the number of output channels in the convolution blocks. The number of output channels of the convolution blocks strongly affects the number of weight parameters of MM-CNN. Therefore, we introduce a hyperparameter *c* for the number of output channels in the convolution blocks. Note that the number of output channels for Conv5 is independent of *c*. The larger *c* is, the larger the number of weight parameters, resulting in the larger scale of MM-CNN. Table 3 shows the configuration of one CNN consisting of MM-CNN when *c* is introduced in the output channel of the convolution blocks.

**Figure 6:**
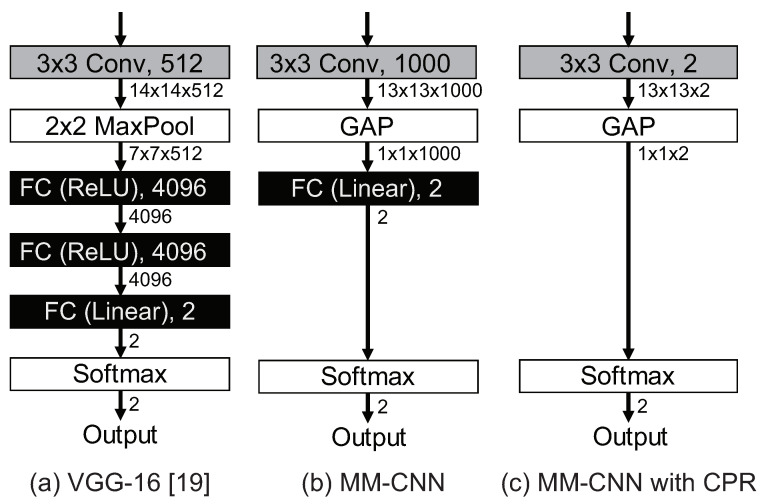
Configuration of CNN classifiers for two-class classification: (**a**) VGG-16 [19], (**b**) MM-CNN, and (**c**) MM-CNN with CPR.

The second approach is to reduce the number of weight parameters by eliminating the fully connected layers without sacrificing the estimation accuracy. Early CNNs such as AlexNet [20] and VGG [19] used three fully connected layers in the classifier, as shown in Figure 6a, where the number of outputs is set to 2 for two-class classification based on whether an attribute is available or not. In general, the number of weight parameters of CNN increases significantly as the number of fully connected layers increases. Recent CNNs such as ResNet [22] and MobileNet [32] reduce the number of weight parameters by using Global Average Pooling (GAP) and one fully connected layer in the classifier, as shown in Figure 6b. The same configuration is used in MM-CNN. However, this configuration is proposed to be used for ImageNet [33] with 1000-class classification. The weight parameters in the classifier can be further reduced, since face attribute estimation is based on two-class classification, which is a simpler task than 1000-class classification. We assume that feature extraction in convolution blocks already classifies the face image into two classes and propose Convolutinalization for Parameter Reduction (CPR) that eliminates all the fully connected layers in the classifier. The configuration of the classifier using CPR is shown in Figure 6c. The number of output channels of Conv5 is set to 2, and the feature map output from Conv5 is aggregated by GAP to obtain two channels of output. The final output is the score obtained by applying the softmax function without passing through a fully connected layer. Some CNNs without fully connected layers have already been proposed such as FCN [34], U-Net [35], MobileNetV2 [36], and EfficientNet [37]. FCN and U-Net are designed for image segmentation, which consist of an encoder and a decoder. The encoder is the same as a feature extractor of general CNNs for image classification, and the fully connected layers are replaced by a decoder including transposed convolution layers to output 2D or 3D matrices. MobileNetV2 and EfficientNet are designed for image classification. All the fully connected layers are replaced by 1 × 1 convolution layers for fast and parallel processing with Graphical Processing Units (GPUs). Unlike the above methods, CPR eliminates fully connected layers without replacing them with other layers to reduce the number of weight parameters in the network. To the best of our knowledge, CPR is the first method to eliminate all the fully connected layers with the aim of reducing the number of weight parameters. The effect of reducing the number of weight parameters by CPR is summarized in Table 4. CPR reduces the number of weight parameters in MM-CNN by 82.4% for Mean and c=30, and by 97.8% for Concat and c=3, respectively. The effect of CPR on reducing the number of parameters in Add and Mean is the same. The effect of CPR in Concat is more significant than that in Add and Mean, since many weight parameters are required in Conv5.

## 5. Experiments and Discussion

In this section, we describe the performance evaluation of the proposed method and ten conventional methods on two public datasets: CelebA [9] and LFW-a [16].

### 5.1. Dataset

**CelebA** (http://mmlab.ie.cuhk.edu.hk/projects/CelebA.html, accessed on 5 September 2019)—This dataset consists of 202,599 face images of 10,177 identities, 40 binary facial attributes, and 5 landmark coordinates. In this experiment, we use face images aligned based on the coordinates of five landmarks: the left eye, the right eye, the nose, the left edge of the mouth, and the right edge of the mouth.

**LFW-a** (https://talhassner.github.io/home/projects/lfwa/, accessed on 17 November 2019)—This dataset consists of 13,233 face images of 5749 identities and 73 binary facial attributes. In the experiment, we use only the 40 facial attributes common to CelebA. We also use face images aligned based on the coordinates of three landmarks: the right eye, the left eye, and the center of the mouth.

### 5.2. Experimental Condition

As for CelebA, 182,637 images and the remaining 19,962 images are used for training and test, respectively. The splitting of the dataset follows the experimental protocol recommended by CelebA. As for LFW-a, 6263 images and the remaining 6880 images are used for training and test, respectively. For both datasets, 10% of the training data is used as validation data to verify overfitting. The cross-entropy loss is used as the loss function in training, and Nesterov Accelerated Gradient (NAG) [38] is used as the optimizer. The initial value of the learning rate is set to 0.025. The maximum number of epochs is set to 50. The batch size is set to 64. If the loss to validation data is not improved for two consecutive epochs, the learning rate is reduced to half. If the loss is not improved in five consecutive epochs, the training is completed. The pixel values of input images are normalized to have 0 mean and 1 variance, are randomly horizontally flipped, and are resized to 227 × 227 pixels. The weight parameters of all convolution layers and fully connected layers are initialized using He initialization [39]. Python 3.8.8 (https://www.python.org, accessed on 1 February 2022), Pytorch 1.8.1 [40], CUDA 10.2 (https://developer.nvidia.com/cuda-toolkit, accessed on 1 February 2022), and cuDNN 7.6.5 (https://developer.nvidia.com/cudnn, accessed on 1 February 2022) are used in the implementation. All the CNN models are trained and evaluated on NVIDIA GeForce GTX 1080 Ti (https://www.nvidia.com/en-us/geforce/10-series/, accessed on 1 February 2022) hardware.

We compare the performance of MM-CNN with that of the ten conventional methods: LNets + ANet [9], FaceNet [10], MT-RBMs [28], MCNN-AUX [2], ATNet_GT [13], PS-MCNN-LC [15], AlexNet + CSFL [14], ABN [21], VGG16 + Auglabel [23], and DeepID2 + CLMLE [27]. We also evaluate the performance of MM-CNN with three merging functions: Concat, Mean, and Add. The performance of MM-CNN is evaluated for Concat at c={1,2,3,4} and for Mean and Add at c={5,10,20,30,60}, respectively. Each method is evaluated on the estimation accuracy of each face attribute or the average of them. In face attribute estimation, each attribute is estimated regarding whether the input face image includes it or not. The estimation accuracy of each attribute is calculated by estimating the attribute for all face images in the test dataset and comparing the estimation results to the ground-truth labels in the test dataset. Note that the average of the estimation accuracy is an average of the estimation accuracy for each attribute after rounding to the third decimal place. In the experimental results, the average of the estimation accuracy is presented except when the estimated accuracy for an attribute index is presented.

### 5.3. Evaluation of Merging Functions and CPR in MM-CNN

**Figure 7:**
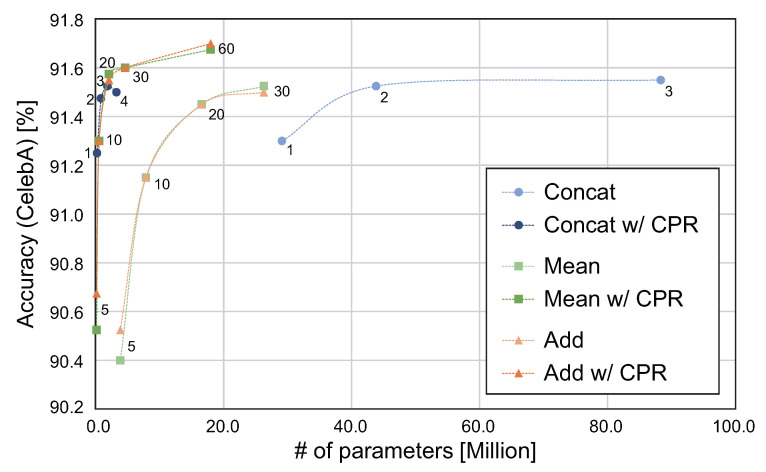
Comparison of the parameter efficiency of MM-CNN with different merge functions and *c*. The numbers near each point in the graph indicate the hyperparameter *c*.

We first evaluate the impact of the merging functions and CPR in MM-CNN for each hyperparameter *c*. Table 5 summarizes the accuracy of face attribute estimation and the number of weight parameters for each dataset when changing the merging functions, *c*, and CPR. “N/A” means that attribute estimation cannot be done due to exceeding the maximum memory size of GPU. Figure 7 shows the trade-off plot between estimation accuracy for CelebA and the number of parameters when varying the merging function, *c*, and CPR used in MM-CNN. The horizontal axis indicates the number of weight parameters, and the vertical axis indicates the average of the estimation accuracy for CelebA, where the estimation accuracy is the average of the estimation accuracy of the 40 attributes. In MM-CNN without CPR, Mean and Add exhibit higher parameter efficiency than Concat. In MM-CNN with CPR, the number of parameters is much smaller than that without CPR. Surprisingly, CPR slightly improves the accuracy of face attribute estimation in MM-CNN. This result suggests that a classifier with many weight parameters, such as fully connected layers, is not effective for a simple binary classification task. CPR is extremely effective in improving the parameter efficiency of MM-CNN, and it also makes optimization easier by reducing the complexity of MM-CNN. In particular, CPR can improve the parameter efficiency of MM-CNN using Concat, since most of the weight parameters are in the fully connected layers, as shown in Table 4. The balance between the number of weight parameters and accuracy of MM-CNN can be adjusted by changing the combination of merging functions, *c*, and CPR. MM-CNN using {Mean, c=20, CPR} and {Concat, c=4, CPR} achieve high-parameter efficiency for CelebA and LFW-a, respectively.

### 5.4. Evaluation of the Number of Parallels in MM-CNN

**Figure 8:**
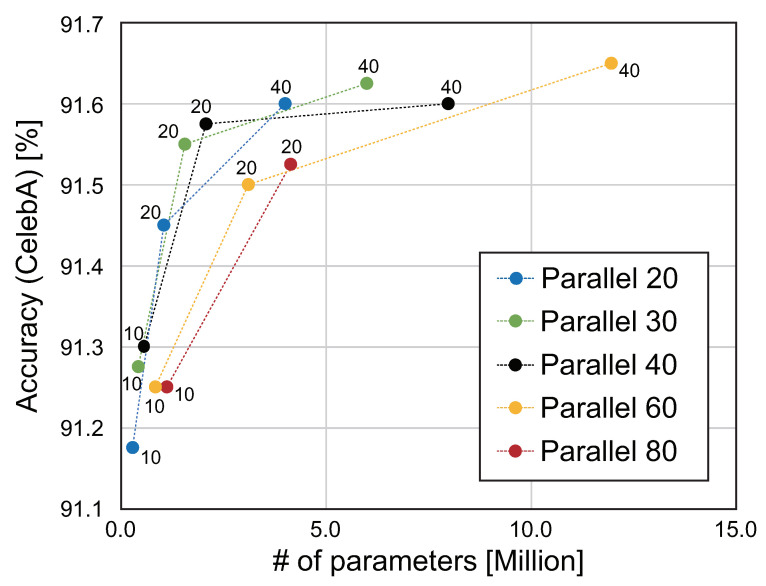
Comparison of the parameter efficiency of MM-CNN using Mean and CPR with the different number of parallels for CelebA. The numbers near each point in the graph indicate the hyperparameter *c*.

As mentioned in Section 4.1. MM-CNN consists of the combination of single-task CNNs. Although the number of single-task CNNs in MM-CNN is set to 40, which is the same as the number of face attributes, the number of parallel networks can be changed. Through this experiment, we verify the number of parallels with high parameter efficiency in MM-CNN. Note that regardless of the number of parallels, the network architecture from Conv5 to FC in Figure 4b is not changed to output 40 scores. The accuracy of face attribute estimation for CelebA and the number of parameters when changing the number of parallels and *c* are summarized in Table 6 and Figure 8, where we use Mean and CPR for all the settings. Note that “N/A” in Table 6 indicates that attribute estimation is not performed, since the maximum memory size of the GPU is exceeded. The parameter efficiency for MM-CNN with 20 and 30 parallels is almost the same as 40 parallels. The above results indicate that the performance of MM-CNN can be maximized with a simple criterion that the number of parallels of MM-CNN is set to be the same as the number of face attributes. On the other hand, the parameter efficiency becomes lower for MM-CNNs with more than 60 parallels. In MM-CNN with Mean and Add, the feature maps extracted from each convolution block are added in each channel. As the number of parallels increases, the information is compressed by the addition of feature maps, resulting in a decrease in estimation accuracy. The estimation accuracy in MM-CNN with Concat will also be reduced, since the next convolution block after merging compresses the information in a similar way.

### 5.5. Comparison with Multi-CNN

We compare the accuracy of face attribute estimation using Multi-CNN and MM-CNN to verify the effectiveness of the merging layer. Multi-CNN uses independent CNNs to estimate each attribute as shown in Figure 4a. Table 7 shows the results of evaluating the estimation accuracy of Multi-CNN and MM-CNN for CelebA by changing *c* and with/without CPR, where we use Mean for MM-CNN. Note that the existence of the merging layers has little effect on the number of weight parameters, except for MM-CNN with Concat. The experimental results show that MM-CNN has higher estimation accuracy than Multi-CNN in all settings. The merging layers can improve the multi-task performance of CNNs with little increase in the number of weight parameters.

### 5.6. Comparison with Conventional Methods

**Figure 9:**
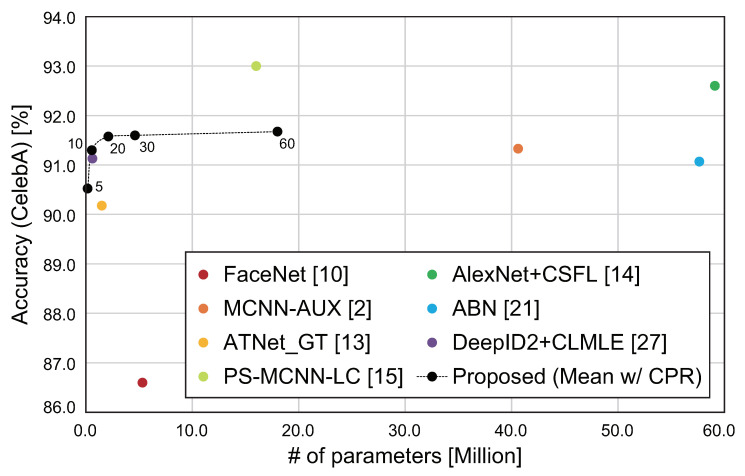
Comparison of the parameter efficiency of face attribute estimation methods. The numbers near each point in the graph indicate the hyperparameter *c*.

We compare the performance of MM-CNN with ten conventional methods: LNets + ANet [9], FaceNet [10], MT-RBMs [28], MCNN-AUX [2], ATNet_GT [13], PS-MCNN-LC [15], AlexNet + CSFL [14], ABN [21], VGG16 + Auglabel [23], and DeepID2 + CLMLE [27]. In this experiment, we use MM-CNN with Mean and focus on the three patterns exhibiting high parameter efficiency from Table 5. We also use MM-CNN with Concat and CPR, which exhibited the highest estimation accuracy for LFW-a in Table 5. Table 8 and Table 9 show the experimental results for CelebA and LFW-a, respectively. Figure 9 shows the parameter efficiency of each method in face attribute estimation. Note that some conventional methods are listed and plotted only on one side in Table 8 and Table 9 and Figure 9. The accuracy of the conventional methods is referred to those described in the paper. If the accuracy for each attribute was not listed or the accuracy for one dataset was not listed such as ABN [21] and DeepID2 + CLMLE [27], the accuracy for those methods is not listed in Table 8 and Table 9. Since SVM-based methods such as LNets + ANet [9] and MT-RBMs [28] cannot evaluate the number of weight parameters, only methods without SVM are plotted in Figure 9.

In CelebA, the accuracy of MCNN-AUX [2] and MM-CNN (Mean, c=10, CPR) is comparable, while the number of parameters of MM-CNN is 1/70 that of MCNN-AUX. Comparing ATNet_GT [13] and MM-CNN (Mean, c=10, CPR), MM-CNN (Mean, c=10, CPR) has 1% higher accuracy and 1/3 of the number of parameters. The accuracy of PS-MCNN-LC [15] and AlexNet + CSFL [14] is higher than MM-CNN, while the number of parameters of MM-CNN with CPR is much smaller than those of them. As mentioned above, MM-CNN exhibited the best parameter efficiency among the compared methods. In addition, since MM-CNN is a network architecture for multi-task processing, the architecture and CPR of MM-CNN can be used in combination with multi-label methods such as concatenating multiple attribute labels.

## 6. Conclusions

In this paper, we proposed a face attribute estimation method using Merged Multi-CNN (MM-CNN), which consists of multiple CNNs in parallel with the merging layers. We also proposed a parameter reduction method called Convolutionalization for Parameter Reduction (CPR), which removes all fully connected layers from MM-CNNs. Through a set of experiments to evaluate the performance on CelebA [9] and LFW-a [16], we demonstrated that MM-CNN can estimate face attributes with high accuracy using CNN with fewer weight parameters than conventional methods. Although the MM-CNN discussed in this paper was based on simple networks, the approach can be applied to recent complex networks. Future work will include extending and improving the accuracy of MM-CNN, applying it to practical applications, and comparing its performance other than face attribute estimation with general multi-task learning methods.

## Figures and Tables

**Table 1:** A summary of face attribute estimation methods.

Method	Feature Extraction	Classifier
Kumar et al. [6]	Pixel value (gray, RGB and HSV), edge magnitude and orientation	One SVM for each attribute
Zhang et al. [8]	Pose Aligned Networks (4 Conv and 1 FC) for Deep Attribute modeling (PANDA)	One linear SVM for each attribute
Liu et al. [9]	LNet (5 Conv) for face localization and ANet (4 Conv) for face attribute prediction	One linear SVM for each attribute
Zhong et al. [10]	FaceNet [18] or VGG-16 [19]	Softmax classifier or one linear SVM for each attribute
Wang et al. [12]	Siamese network (2 Conv and 7 Inception and 1 Fc)	Softmax
Hand et al. [2]	Multi-task deep Convolutional Neural Network (MCNN) (3 Conv and 2 FC)	Softmax classifier with an AUXilirary network (AUX)
Gao et al. [13]	ATNet, ATNet_G, ATNet_GT (4 Conv and 3 FCc)	Softmax
Cao et al. [15]	Partially Shared MCNN (PS-MCNN) (5 Conv and 2 FCc)	Multi-label classifier
Han et al. [14]	AlexNet-like CNN [20] (5 Conv and 4 FC) with facial landmark detector	Multi-label classifier
Fukui et al. [21]	Attention Branch Network (ABN) based on ResNet-101 [22]	Softmax
Bhattarai et al. [23]	VGG-16 [19] and word2vec [24]	Multi-label classifier
Chen et al. [25]	Hard Parameter Sharing-Channel Split network (HPS-CS) based on AlexNet (9 Conv and 1 FC)	Softmax
Huang et al. [11]	DeepID2 [26]	Large Margin Local Embedding (LMLE)-kNN
Huang et al. [27]	ResNet-like CNN (64 Conv and 1 FC) with facial landmark detector	Cluster-based Large Margin Local Embedding (CLMLE)
Ehrlich et al. [28]	Multi-Task Restricted Boltzmann Machines (MT-RBMs) with PCA with facial landmark detector	Softmax

**Table 2:** Face attribute labels defined in CelebA [9].

Idx.	Attribute	Idx.	Attribute
1	5 O’Clock Shadow	21	Male
2	Arched Eyebrows	22	Mouth Slightly Open
3	Attractive	23	Mustache
4	Bags Under Eyes	24	Narrow Eyes
5	Bald	25	No Beard
6	Bangs	26	Oval Face
7	Big Lips	27	Pale Skin
8	Big Nose	28	Pointy Nose
9	Black Hair	29	Receding Hairline
10	Blond Hair	30	Rosy Cheeks
11	Blurry	31	Sideburns
12	Brown Hair	32	Smiling
13	Bushy Eyebrows	33	Straight Hair
14	Chubby	34	Wavy Hair
15	Double Chin	35	Wearing Earrings
16	Eyeglasses	36	Wearing Hat
17	Goatee	37	Wearing Lipstick
18	Gray Hair	38	Wearing Necklace
19	Heavy Makeup	39	Wearing Necktie
20	High Cheekbones	40	Young

**Table 3:** Configuration of one CNN consisting of MM-CNN when *c* is introduced in the output channel of the convolution blocks.

Layer	Kernel	Stride	Padding	Output Shape
Conv 1	7×7	4	2	56×56×c
BatchNorm 1				56×56×c
MaxPool 1	3×3	2	0	28×28×c
Conv 2	5×5	1	1	28×28×(2×c)
BatchNorm 2				28×28×(2×c)
MaxPool 2	3×3	2	0	12×12×(2×c)
Conv 3	3×3	1	1	12×12×(2×c)
BatchNorm 3				12×12×(2×c)
Conv 4	3×3	1	1	12×12×(2×c)
BatchNorm 4				12×12×(2×c)
Conv 5	3×3	1	1	12×12×1000
BatchNorm 5				12×12×(2×c)
MaxPool 3	3×3	2	0	5×5×1000
GAP				1×1×1000
FC				2

**Table 4:** Effect of reducing the number of weight parameters by CPR, where “Ratio” indicates the ratio of the number of weight parameters in each conv block to the total number of weight parameters in the MM-CNN.

Type	MM-CNN (Mean, c=30)			
	w/o CPR		w/ CPR	
	# of Params	Ratio	# of Params	Ratio
Conv1	176,400	0.7%	176,400	3.8%
Conv2	1,800,000	6.9%	1,800,000	39.0%
Conv3	1,296,000	4.9%	1,296,000	28.1%
Conv4	1,296,000	4.9%	1,296,000	28.1%
Conv5	21,600,000	82.3%	43,200	1.0%
FC	80,080	0.3%	—	—
Total	26,248,480	100%	4,611,600	100%
**Type**	**MM-CNN (Concat, c=3)**			
	**w/o CPR**		**w/ CPR**	
	**# of Params**	**Ratio**	**# of Params**	**Ratio**
Conv1	17,640	0.1%	17,760	0.9%
Conv2	720,000	0.8%	720,240	37.0%
Conv3	518,400	0.6%	518,640	26.6%
Conv4	518,400	0.6%	518,640	26.6%
Conv5	86,400,000	97.8%	172,800	8.9%
FC	80,080	0.1%	—	—
Total	88,254,520	100%	1,947,240	100%

**Table 5:** Accuracy of face attribute estimation and the number of parameters on both datasets when changing the merging functions, *c*, and CPR of MM-CNN, where “N/A” means that attribute estimation cannot be done due to exceeding the maximum memory size of GPU. Best accuracy is shown with underline.

Merging Function	*c*	w/o CPR			w/ CPR		
		CelebA	LFW-a	Params	CelebA	LFW-a	Params
Concat	1	91.30%	84.90%	29.12 M	91.25%	85.85%	0.26 M
	2	91.53%	85.90%	58.51 M	91.48%	86.10%	0.91 M
	3	91.55%	85.50%	88.30 M	91.53%	86.15%	1.95 M
	4	N/A	N/A	118.48 M	91.50%	86.33%	3.27 M
Mean	5	90.40%	81.70%	3.87 M	90.53%	78.80%	0.16 M
	10	91.15%	82.65%	7.87 M	91.30%	84.48%	0.56 M
	20	91.45%	83.45%	16.60 M	91.58%	85.28%	2.10 M
	30	91.53%	84.95%	26.30 M	91.60%	85.10%	4.62 M
	60	N/A	N/A	61.26 M	91.68%	85.54%	18.02 M
Add	5	90.53%	78.55%	3.87 M	90.68%	83.35%	0.16 M
	10	91.15%	82.60%	7.87 M	91.30%	83.45%	0.56 M
	20	91.45%	82.98%	16.60 M	91.55%	85.25%	2.10 M
	30	91.50%	82.58%	26.30 M	91.60%	85.05%	4.62 M
	60	N/A	N/A	61.26 M	91.70%	85.15%	18.02 M

**Table 6:** Estimation accuracy of MM-CNN with Mean and CPR under varying the number of parallels for CelebA.

# of Parallels	*c*	Accuracy	Params
20	10	91.18%	0.29 M
	20	91.45%	1.06 M
	40	91.60%	4.06 M
30	10	91.28%	0.43 M
	20	91.55%	1.58 M
	40	91.63%	6.08 M
40	10	91.30%	0.57 M
	20	91.58%	2.10 M
	40	91.60%	8.10 M
60	10	91.25%	0.85 M
	20	91.50%	3.15 M
	40	91.65%	12.14 M
80	10	91.25%	1.13 M
	20	91.53%	4.20 M
	40	N/A	16.18 M

**Table 7:** Estimation accuracy Multi-CNN and MM-CNN with Mean for CelebA. ✓ shows that CPR is used. Best accuracy is shown with underline.

CPR	*c*	Multi-CNN	MM-CNN
	5	89.73%	90.40%
	10	90.33%	91.15%
	20	90.80%	91.45%
	30	90.90%	91.53%
✓	5	90.00%	90.53%
	10	90.45%	91.30%
	20	90.95%	91.58%
	30	91.03%	91.60%
	60	91.15%	91.68%

**Table 8:** Estimation accuracy of face attribute estimation methods for CelebA. Best accuracy is shown with underline.

Method	Attribute Index										
	1	2	3	4	5	6	7	8	9	10	
LNet + ANet [9]	91	79	81	79	98	95	68	78	88	95	
FaceNet [10]	89	83	82	79	96	94	70	79	87	93	
MT-RBMs [28]	90	77	76	81	98	88	69	81	76	91	
MCNN-AUX [2]	95	83	83	85	99	96	71	85	90	96	
ATNet_GT [13]	92	81	81	84	99	96	71	83	89	95	
PS-MCNN-LC [15]	97	86	84	87	99	98	73	86	92	98	
AlexNet + CSFL [14]	95	86	85	99	99	96	88	92	85	91	
MM-CNN (Concat, c=4, CPR)	94	84	83	85	99	96	72	85	90	96	
MM-CNN (Mean, c=60, CPR)	95	84	83	86	99	96	72	85	91	96	
MM-CNN (Mean, c=30, CPR)	95	84	83	86	99	96	72	85	90	96	
MM-CNN (Mean, c=10, CPR)	95	84	83	86	99	96	71	84	90	96	
**Method**	**Attribute Index**										
	**11**	**12**	**13**	**14**	**15**	**16**	**17**	**18**	**19**	**20**	
LNet+ANet [9]	84	80	90	91	92	99	95	97	90	87	
FaceNet [10]	87	79	87	88	89	99	94	95	91	87	
MT-RBMs [28]	95	83	88	95	96	96	96	97	85	83	
MCNN-AUX [2]	96	89	93	96	96	100	97	98	92	88	
ATNet_GT [13]	96	87	92	94	96	99	97	98	90	86	
PS-MCNN-LC [15]	98	91	95	98	98	100	98	99	93	89	
AlexNet + CSFL [14]	96	96	85	97	99	99	98	96	92	88	
MM-CNN (Concat, c=4, CPR)	96	89	93	96	97	100	97	98	92	88	
MM-CNN (Mean, c=60, CPR)	96	90	93	96	97	100	98	98	92	88	
MM-CNN (Mean, c=30, CPR)	96	90	93	96	97	100	97	98	92	88	
MM-CNN (Mean, c=10, CPR)	96	89	93	96	97	100	97	98	92	88	
**Method**	**Attribute Index**										
	**21**	**22**	**23**	**24**	**25**	**26**	**27**	**28**	**29**	**30**	
LNet + ANet [9]	98	92	95	81	95	66	91	72	89	90	
FaceNet [10]	99	92	93	78	94	67	85	73	87	88	
MT-RBMs [28]	90	82	97	86	90	73	96	73	92	94	
MCNN-AUX [2]	98	94	97	87	96	76	97	77	94	95	
ATNet_GT [13]	97	93	97	86	94	76	97	75	93	95	
PS-MCNN-LC [15]	99	96	99	89	98	77	99	79	96	97	
AlexNet+CSFL [14]	98	94	97	90	97	78	97	78	94	96	
MM-CNN (Concat, c=4, CPR)	98	94	97	88	96	76	97	78	94	95	
MM-CNN (Mean, c=60, CPR)	98	94	97	88	96	77	97	78	94	96	
MM-CNN (Mean, c=30, CPR)	98	94	97	88	96	76	97	78	94	96	
MM-CNN (Mean, c=10, CPR)	98	94	97	88	96	76	97	77	94	95	
**Method**	**Attribute Index**										
	**31**	**32**	**33**	**34**	**35**	**36**	**37**	**38**	**39**	**40**	**Ave.**
LNet + ANet [9]	96	92	73	80	82	99	93	71	93	87	87.3
FaceNet [10]	95	92	73	79	82	96	93	73	91	86	86.6
MT-RBMs [28]	96	88	80	72	81	97	89	87	94	81	87.0
MCNN-AUX [2]	98	93	84	84	90	99	94	87	97	88	91.3
ATNet_GT [13]	97	92	80	82	89	99	93	86	96	88	90.2
PS-MCNN-LC [15]	98	95	86	86	93	99	96	89	99	91	93.0
AlexNet + CSFL [14]	98	94	85	87	91	99	93	89	97	90	92.6
MM-CNN (Concat, c=4, CPR)	98	93	84	84	91	99	94	88	97	89	91.5
MM-CNN (Mean, c=60, CPR)	98	93	84	84	91	99	94	88	97	89	91.7
MM-CNN (Mean, c=30, CPR)	98	93	84	84	91	99	94	88	97	89	91.6
MM-CNN (Mean, c=10, CPR)	98	93	83	83	90	99	94	87	97	88	91.3

**Table 9:** Estimation accuracy of face attribute estimation methods for LFW-a. Best accuracy is shown with underline.

**Method**	Attribute index										
	**1**	**2**	**3**	**4**	**5**	**6**	**7**	**8**	**9**	**10**	
LNet + ANet [9]	84	82	83	83	88	88	75	81	90	97	
FaceNet [10]	77	83	79	83	91	91	78	83	90	97	
MCNN-AUX [2]	77	82	80	83	92	90	79	85	93	97	
PS-MCNN-LC [15]	88	84	82	87	93	91	83	86	93	98	
AlexNet + CSFL [14]	80	86	84	92	93	77	81	80	83	91	
MM-CNN (Concat, c=4, CPR)	78	81	81	83	93	92	79	84	92	97	
MM-CNN (Mean, c=60, CPR)	77	80	80	82	93	91	77	83	92	97	
MM-CNN (Mean, c=30, CPR)	76	80	80	82	92	90	76	83	92	97	
MM-CNN (Mean, c=10, CPR)	76	79	80	81	92	90	74	83	92	97	
**Method**	**Attribute Index**										
	**11**	**12**	**13**	**14**	**15**	**16**	**17**	**18**	**19**	**20**	
LNet + ANet [9]	74	77	82	73	78	95	78	84	95	88	
FaceNet [10]	88	76	83	75	80	91	83	87	95	88	
MCNN-AUX [2]	85	81	85	77	82	91	83	89	96	88	
PS-MCNN-LC [15]	87	82	86	78	87	93	84	91	97	89	
AlexNet + CSFL [14]	75	97	82	78	92	86	88	89	95	89	
MM-CNN (Concat, c=4, CPR)	85	82	85	76	82	92	84	89	95	87	
MM-CNN (Mean, c=60, CPR)	85	82	83	76	80	91	83	89	95	87	
MM-CNN (Mean, c=30, CPR)	85	82	83	75	80	90	83	89	95	86	
MM-CNN (Mean, c=10, CPR)	84	81	82	74	79	89	82	88	94	86	
**Method**	**Attribute Index**										
	**21**	**22**	**23**	**24**	**25**	**26**	**27**	**28**	**29**	**30**	
LNet + ANet [9]	94	82	92	81	79	74	84	80	85	78	
FaceNet [10]	94	81	94	81	80	75	73	83	86	82	
MCNN-AUX [2]	94	84	93	83	82	77	93	84	86	88	
PS-MCNN-LC [15]	95	85	94	84	82	78	95	88	87	89	
AlexNet + CSFL [14]	93	86	95	82	81	75	91	84	85	86	
MM-CNN (Concat, c=4, CPR)	94	82	94	82	81	79	91	85	87	87	
MM-CNN (Mean, c=60, CPR)	93	80	93	79	79	76	91	83	86	88	
MM-CNN (Mean, c=30, CPR)	93	79	93	78	80	76	90	83	87	87	
MM-CNN (Mean, c=10, CPR)	93	79	93	75	80	75	90	82	86	85	
**Method**	**Attribute Index**										
	**31**	**32**	**33**	**34**	**35**	**36**	**37**	**38**	**39**	**40**	**Ave.**
LNet + ANet [9]	77	91	76	76	94	88	95	88	79	86	83.9
FaceNet [10]	82	90	77	77	94	90	95	90	81	86	84.7
MCNN-AUX [2]	83	92	79	82	95	90	95	90	81	86	86.3
PS-MCNN-LC [15]	84	93	80	83	96	91	96	91	82	87	87.4
AlexNet + CSFL [14]	80	92	79	80	94	92	93	91	81	87	86.0
MM-CNN (Concat, c=4, CPR)	84	91	79	82	94	91	95	90	83	85	86.3
MM-CNN (Mean, c=60, CPR)	83	90	79	81	94	90	94	89	82	85	85.5
MM-CNN (Mean, c=30, CPR)	82	90	78	80	94	90	94	89	81	84	85.1
MM-CNN (Mean, c=10, CPR)	82	89	75	80	94	90	94	89	81	84	84.5

## Data Availability

Publicly available datasets were analyzed in this study. The CelebA dataset can be found here: http://mmlab.ie.cuhk.edu.hk/projects/CelebA.html (accessed on 5 September 2019). The LFW-a dataset can be found here: https://talhassner.github.io/home/projects/lfwa/ (accessed on 17 November 2019).
